# A Fresh Look at Problem Areas in Research Methodology in Nutrition

**DOI:** 10.3390/nu17060972

**Published:** 2025-03-10

**Authors:** Norman J. Temple

**Affiliations:** Centre for Science, Athabasca University, Athabasca, AB T9S 3A3, Canada; normant@athabascau.ca

**Keywords:** cohort studies, confounding, mechanistic research, nutrition research, randomized controlled trials, research methods

## Abstract

This paper makes a critical evaluation of several of the research methods used to investigate the relationship between diet, health, and disease. The two widely used methods are randomized controlled trials (RCTs) and prospective cohort studies. RCTs are widely viewed as being more reliable than cohort studies and for that reason are placed higher in the research hierarchy. However, RCTs have inherent flaws and, consequently, they may generate findings that are less reliable than those from cohort studies. The text presents a discussion of the errors that may occur as a result of confounding. This refers to the correlation of the exposure and the outcome with other variables and can mask the true association or produce false associations. Another source of error is reverse causation, which is most commonly associated with cross-sectional studies. These studies do not allow researchers to determine the temporal sequence of lifestyle and other inputs together with health-related outcomes. As a result, it may be unclear which is cause and which is effect. This may also occur with cohort studies and can be illustrated by the inverse association between alcohol intake and coronary heart disease. Mechanistic research refers to the investigation of the intricate details of body functioning in health and disease and this research strategy is widely used in biomedical science. The evidence presented here makes the case that most of our information of practical value in the field of nutrition and disease has come from epidemiological research, including RCTs, whereas mechanistic research has been of minor value.

## 1. Introduction

Many research methods have been developed for investigating the relationship between diet, health, and disease [1]. Each has its advantages and limitations, and new methods are still being developed. Jacobs and Temple [2] describe the most commonly used methods used in nutrition research. The two methods of great importance are randomized controlled trials (RCTs) and prospective cohort studies. Other commonly used methods include case-control studies, cross-sectional studies, and population studies (ecological studies).

There are numerous sources of error that commonly appear in research studies. Of particular importance is the estimation of the usual food intake of study participants, which is prone to significant error that can sometimes lead to misleading results [3,4]—a topic that has been repeatedly discussed. With case-control studies there are significant sources of error above and beyond those that occur in cohort studies [2]. One is the “healthy volunteer effect”. This refers to the tendency for health-conscious people who eat a relatively healthy diet to volunteer to be controls (the comparison group for cases). Another error is “recall bias”: the tendency of patients who have recently been diagnosed with a disease, such as cancer, to have a distorted recollection of their past diet and to overstate its unhealthy features. Let us suppose researchers are investigating whether patients with a particular type of cancer have a history of eating, on average, a relatively unhealthy diet compared with persons without cancer. Both of the above sources of error will tend to push the results in the direction of indicating that persons with cancer do eat a less healthy diet, such less fruits and vegetables.

The above sources of error are well known. The subject matter of this paper concerns sources of error that are often overlooked as well as some controversial issues.

## 2. A Comparison of Randomized Controlled Trials and Prospective Cohort Studies in Nutrition Research

The conventional viewpoint is that the results generated by RCTs are inherently more reliable than those coming from cohort studies. Expressed differently, RCTs are placed above cohort studies in the research hierarchy [5]. The rationale for this is seemingly based on sound reasoning. The design of RCTs allows the researchers to carefully control all the conditions in a study so that there is only one difference between the treatment and the control group (e.g., the test group may be given a supplement of zinc). That factor must therefore be the cause of any differences in outcomes between the various groups.

With cohort studies, by contrast, subjects are free to choose their own lifestyle including diet, exercise, alcohol intake, and so forth. For that reason, cohort studies and other epidemiological studies of free-living subjects are referred to as “observational studies”. As a result, any comparison between the subjects for variables of interest, such as the association between intake of vitamin C and risk of cancer, will inevitably have many variables. This can easily lead to false associations.

Clearly, RCTs have several obvious advantages over cohort studies. But a detailed comparison reveals that this is often not the case [6]. Indeed, much evidence suggests that cohort studies can be as reliable as RCTs and may often be even more reliable. This was demonstrated by Temple [7], who made a comparison of eight sets of studies where the same topic was investigated using both RCTs and epidemiological methods (cohort studies in seven of the cases). In each case the objective was to investigate the action of a dietary factor in relation to a disease or other health outcome. He compared the findings from these two types of study.

This detailed comparison revealed that RCTs often have design features that may cause serious errors, whereas cohort studies avoid these problems. The central problem is that RCTs have major practical problems that constrain their design. Suppose the goal is to investigate whether the intervention reduces the risk of the disease under study. In this scenario, it is challenging to find large numbers of healthy volunteers who will agree to follow a modified diet for many years. It is also very costly. In order to overcome these problems, it is necessary to shorten the duration of the follow-up and reduce the number of subjects but still be able to generate statistically significant results. This can be done by recruiting subjects who either have a history of the disease under study or are at high risk of it. In some of the RCTs where the endpoint is a clinical disease, the follow-up period has been as long as 8 or 10 years. But this design greatly limits the value of the study. That is because chronic diseases typically require considerably longer than a decade to develop and often several decades. It may be that the critical period for an intervention to be effective is early in the disease etiology. But an RCT that uses subjects with a history of the disease or at high risk of the disease means that the intervention is occurring late in the disease development. A related problem is that the intervention may need a duration of 10 years or more to be effective. Again, this would mean that in most RCTs the intervention is probably doomed to failure.

Cohort studies circumvent these problems. They typically recruit healthy people free of the disease of interest and track them for between 5 and 15 years. If the study includes a sufficient number of subjects who habitually follow the key diet feature of interest as well as many others who do not, then the study should be able to reveal whether the diet feature is effective (negatively or positively). Moreover, this goal should be achievable irrespective of whether the diet feature acts early or late in the disease history.

RCTs very often measure changes in biomarkers rather than clinical endpoints. This type of study typically has a follow-up of only a few weeks or months. However, it must be stressed that there is always much uncertainty when extrapolating the findings regarding biomarkers into changes in risk of actual clinical disease. By contrast, we can have far more confidence in the findings of a cohort study with a long follow-up and a sufficient number of cases of the disease of interest. RCTs that investigate the effect of diet on blood pressure and body weight resemble the studies of biomarkers with respect to the length of follow-up.

A recent publication discussed the potential errors associated with biomarkers and of other surrogate endpoints [8]. They discussed in detail how this problem may be overcome, at least in part, by the use of reporting guidelines for trials that use surrogate endpoints as the primary outcomes. This checklist is known as the CONSORT (Consolidated standards of reporting trials) [9]. Proposals were recently made for an expansion of this checklist [8].

Another noteworthy advantage of cohort studies is that they allow the study of unhealthy features of the diet. As the subjects have self-selected their diet, a major source of ethical problems is avoided. But in the case of RCTs, of course, it is generally not possible to ask subjects to add substances to their diet that are known to be unhealthy, especially if there is a long follow-up.

In summary, in terms of generating reliable results, RCTs are often superior to cohort studies, but RCTs have inherent flaws which mean that they may often be less reliable than cohort studies. It is essential to carefully evaluate each study based on its design features. We cannot assume that the results of an RCT can be freely applied beyond the specific features of the study.

Schwingshackl et al. [10] carried out a very detailed comparison of RCTs and cohort studies. They made 97 sets of comparison of the findings from the two types of study in the area of diet and disease. Overall, when the comparison involved an identical diet exposure, the two methods produced similar findings. These findings were confirmed in a follow-up study [11] and were consistent with the previous conclusions.

## 3. Problems Caused by Incomplete Reporting

Research publications often omit important details. Incomplete and inadequate reporting of research hampers the assessment of the strengths and weaknesses of the studies reported in the medical literature. The use of a checklist based on CONSORT when reporting the findings of RCTs that use surrogate endpoints was discussed above. Weaver et al. [12] made detailed proposals of how CONSORT can be further developed for use in RCTs in the field of nutrition. Another checklist has been developed for use in observational studies. This is known as STROBE (Strengthening the reporting of observational studies in epidemiology) [13].

## 4. The Challenge of Confounding

While cohort studies are of enormous value, it is important to be aware of their flaws. Of particular importance is that misleading results can arise as a result of confounding. This refers to the correlation of the exposure and the outcome with other variables and can mask the true association or produce false associations. Confounding is a major challenge in cohort studies as illustrated by the following example. The results of a cohort study reveal that persons with a relatively high intake of dietary factor A have a 50% increased risk of developing disease X. However, a closer examination of the data reveals that dietary factor A is associated with smoking which is also associated with disease X. This means that the association between dietary factor A and disease X may be spurious.

Confounding is of great importance in epidemiological studies as lifestyle factors tend to cluster together. Most often this lifestyle clustering takes the form of some people leading a healthy lifestyle with respect to diet, smoking, and exercise, while others lead a generally unhealthy lifestyle. Epidemiologists are well aware of this problem and endeavor to eliminate this source of error in their statistical analysis of the data. However, residual confounding is always possible in observational studies. In summary, confounding can easily lead to false associations (“guilt—or innocence—by association”).

An especially illuminating example of how confounding can lead to highly misleading conclusions is shown by studies of the relationship between beta-carotene and cancer. By the 1980s, much evidence had accumulated that demonstrated a protective association between dietary intake of beta-carotene and the risk of several types of cancer [14]. Dietary intake was estimated based on either the intake of foods that contain beta-carotene or on blood analyses. This evidence came mostly from cohort and case-control studies. These findings were widely interpreted as indicating a cause-and-effect relationship. It was therefore predicted that supplements of beta-carotene would reduce the incidence of cancer. In response to these apparently important findings, several RCTs were carried out but the results were uniformly negative [15].

This begs the question: Why did the findings from observational studies indicate that beta-carotene is protective against cancer whereas RCTs showed no evidence that supplements of the vitamin have any benefit? By far the most plausible explanation lies in confounding which led to a serious error in the interpretation of the findings coming from observational studies. People do not eat beta-carotene; they eat foods rich in the vitamin. Therefore, the correct interpretation of the observational results is that a diet with a relatively high content of foods rich in beta-carotene will probably lower the risk of cancer. As the vitamin comes from fruit and vegetables, this means that fruit and vegetables probably prevent cancer. This has been confirmed many times by cohort studies which have generated strong evidence that fruit and vegetables, especially cruciferous vegetables and green-yellow vegetables, do indeed lower the risk of cancer [16].

Another example of confounding is seen in studies of ultra-processed foods (UPFs). A great weight of evidence has steadily accumulated that demonstrates which foods are associated with increased risk of disease and which enhance health and lower the risk of disease. Advice to the general population regarding which foods to eat is based, to a large degree, on this evidence. Dietary advice also aims to ensure that the diet provides appropriate amounts of all macronutrients and micronutrients. An alternative system classifies foods based on the degree of processing. Foods that are highly processed and that contain artificial additives are classified as UPFs. This classification system disregards the nutrient composition of foods. The system is based on the assumption that all UPFs are unhealthy.

Findings from numerous cohort studies have reported that UPFs are associated with an increased risk of various diseases and poor health outcomes including cardiovascular disease (CVD), type 2 diabetes, common mental disorders (especially depression), and all-cause mortality [17,18,19]. Temple [20] recently proposed how these findings can be best explained. In the cohort studies, UPFs were merged into a single food group regardless of whether the ingredients in each food had a positive or negative association with the risk of disease. However, more detailed analysis reveals that particular types of UPF, such as processed meat and sugar-sweetened beverages, have an especially strong association with disease risk. Conversely, those UPFs comprised of relatively healthy foods have little or no association with disease risk. In the case of the latter foods, the association with disease risk is due to including these foods with unhealthy foods and represents a type of confounding. The total intake of UPF may be acting merely as a crude indicator for the consumption of unhealthy foods.

Confounding can also occur in cohort studies or case-control studies when important variables have been excluded from the analysis of diet-disease associations. Socio-economic status (SES) is an example of this. It is well-established that SES has a significant association with lifestyle, diet, and risk of disease. For that reason, it is usually included in the multivariate analyses. However, a survey of cohort studies reported that one-third of them failed to include any indicator of SES [21]. An additional issue is the measure used for SES. This is usually done in terms of the education of each subject and has the advantage that most people are willing to provide the required information. However, there may be a gap of 20 or 30 years between the end of a person’s education and their enrolment in the cohort study. Because of this, education may not be a reliable indicator of current SES. A person’s current income (or family income) or social class may be a better indicator, but these are seldom determined. Whether this is a source of significant error has not been properly investigated.

Maki et al. [3] identified another case where there was a failure to measure an important variable in cohort studies of diet–disease associations. Cardiorespiratory fitness and physical activity are strong predictors of CVD risk. However, most large cohort studies that investigated the relationship between diet and CVD risk did not measure cardiorespiratory fitness. As in the previous example, this can potentially lead to error caused by confounding.

A recent development in the area of observational studies is the use of directed acyclic graphs (DAGs) [22]. DAGs are increasingly popular in applied health research as a transparent means of identifying confounding variables that require conditioning to estimate causal effects.

## 5. Reverse Causation as a Cause of Misleading Conclusions

Many cross-sectional studies have revealed an association between, on the one hand, dietary factors or other lifestyle variables and, on the other hand, the presence of ill health. Such findings suggest possible cause-and-effect relationships. However, by their very nature cross-sectional studies do not allow researchers to determine the temporal sequence of lifestyle and other inputs and health-related outcomes. In most such cases there is little doubt as to which is cause and which is effect. For example, a cross-sectional study may reveal that residents of the northern states of the USA tend to have a relatively low blood level of vitamin D. In this case there is little doubt that latitude of residence is the cause of the low blood level of vitamin D, but in many cases the temporal relationship is unclear, and this makes it equally unclear which is cause and which is effect. This may occur if it is possible that the change in the health-related outcome leads to the change in diet or lifestyle. This is often referred to as reverse causation.

The following study illustrates how this confusion can occur. Meng and D’Arcy [23] carried out a cross-sectional study and observed an inverse association between physical activity and the presence of various mental disorders. This begs the question: Which was cause and which was effect? The most plausible explanation for these findings is as follows. While a low level of exercise may, to some extent, increase the risk of mental disorders, it is far more likely that persons with mental disorders often lose the desire to engage in exercise [24]. Curiously, the authors interpreted their findings as indicating that physical inactivity is an important cause of mental disorders. They then went one step further and estimated the number of cases of mental disorders that could be prevented if more people engaged in physical activity.

It would be a mistake to assume that cohort studies are somehow immune to the problem of reverse causation. The relationship between alcohol and coronary heart disease (CHD) provides an example of this. Many cohort studies have reported that a low to moderate consumption of alcohol has a protective association with the risk of CHD [25]. The direction of cause and effect appears obvious. Indeed, for many years, there was a clear consensus that persons who consume alcohol in moderation are at reduced risk of CHD. However, there has been much controversy in the interpretation of this evidence. The focus here is on abstainers from alcohol. The majority of cohort studies make the assumption that abstainers are typical of the general population except that they choose not to consume alcohol. However, this assumption is flawed as it ignores the fact that many people quit drinking after they develop a health problem. People with this history are often at increased risk of CHD. As a result, these “sick quitters” may have a higher risk of CHD than moderate drinkers. This is a form of reverse causation as it is not alcohol that prevents CHD but rather that being at an increased risk of CHD causes people to quit drinking.

This hypothesis was proposed by Shaper and colleagues [26] and was given support by an analysis of cohort studies carried out by Fillmore et al. [27]. This phenomenon is also seen for the relationship between alcohol consumption and all-cause mortality [28]. These findings strongly suggest that alcohol consumption in moderation provides very little if any protection against CHD or all-cause mortality.

## 6. The Limitations of Mechanistic Research

Mechanistic research refers to the investigation of the intricate details of body functioning in health and disease, and it is often referred to as reductionism [29]. This research strategy constitutes a substantial part of the research being carried out into the causes of disease and the search for improved means for preventing and treating disease. As a research strategy, mechanistic research appears to make good sense. If we can more fully understand how the body works and the malfunctions that have occurred when a disease strikes, then we should be able to effectively prevent and treat disease. This strategy may be likened to a car mechanic who fully understands how cars work and applies that knowledge to repairing cars that have broken down. In some areas this strategy has achieved much success. A notable example is drug development: many important drugs owe their discovery to mechanistic research [30].

A very different story emerges, however, when we look at research in the field of nutrition and disease [30,31]. Most of our information of practical value has come from epidemiological research, including RCTs, whereas mechanistic research has been of limited value. This will be illustrated with several examples.

### 6.1. Carbohydrates, Fat, and Weight Gain

For decades there has been an enormous amount of research and endless debate regarding the optimal balance between dietary carbohydrates and fat for preventing excess weight gain and for achieving weight loss. A large part of this research can be categorized as mechanistic research, such as studies of the actions of different hormones and of intermediary metabolism. But translating this work into dietary advice that survives testing in the real world has been notably unsuccessful. The proof of the pudding is in the eating but in this area mechanistic research does not tell us how to design a pudding for weight control. By contrast, the majority of our information of practical value has been generated by cohort studies and RCTs. Of particular importance, RCTs suggest that diets with a reduced content of carbohydrates are generally more effective for achieving weight loss than are low-fat diets [32].

Other lines of investigation into the relationship between diet and weight control point to the same conclusion. Findings from cohort studies and RCTs demonstrated that intake of sugar-sweetened beverages is associated with weight gain [33]. By contrast, cohort studies and RCTs indicate that nuts are protective against weight gain [34]. RCTs have reported that the Mediterranean diet can be effective in achieving weight loss [35]. In each of these cases it is highly doubtful if mechanistic research would have succeeded in generating these valuable findings.

### 6.2. Diet and Coronary Heart Disease

Many thousands of research studies have been carried out since the 1960s into the relationship between diet and CHD. For about 50 years, from roughly 1974 to 2014, the dominant causal dietary factor was believed to be saturated fat [36]. The supposed mechanism was as follows: the diet typically eaten in Western countries has an excess content of both fat and saturated fat which leads to a high blood cholesterol level and thence to atherosclerosis and CHD. By around 2014, it had become increasingly clear that saturated fat has only a weak association with risk of CHD. At the same time much evidence demonstrated that other components of the diet have a stronger (positive or negative) association [36]. Sugar-sweetened beverages and trans fats increase risk whereas whole grain cereals, fish, and fruit and vegetables are protective [36].

The majority of the supporting evidence for the above came from cohort studies. Of particular note, Willett and colleagues at Harvard University carried out large cohort studies and published dozens of papers on the relationship between diet and CHD, e.g., [37,38]. Valuable evidence has also come from RCTs. For example, findings from RCTs reveal that blood pressure can be reduced by an increased intake of fruit juice [39] or a reduced intake of sodium [40]. Mechanistic studies, in stark contrast, have been of minimal value. This is demonstrated by the fact that review papers that explore the relationship between diet and risk of CHD focus on epidemiological studies, including RCTs, but seldom include research findings that come from studies of the coronary artery during the development of atherosclerosis.

### 6.3. Vitamin D

For several decades vitamin D was synonymous with calcium balance and bone health. However, in recent years an impressive body of evidence has emerged that strongly indicates that a low blood level of the vitamin is associated with an increased risk of an assortment of health disorders, and that supplements may prevent at least some of these disorders [41,42,43,44]. These findings came overwhelmingly from cohort studies and RCTs, while mechanistic studies played a mere incidental role.

### 6.4. Anthocyanins

Many cohort studies have reported a protective association between intake of fruit and vegetables and a wide variety of diseases. More detailed analysis reveals that anthocyanins appear to be of particular importance. These are a class of phytochemicals found in berries, grapes, and some vegetables. Based on these findings many RCTs were carried out in which subjects were given supplements of either purified anthocyanins or of foods rich in these substances. The findings from the RCTs and cohort studies strongly indicate that anthocyanins are protective against type 2 diabetes while also bringing about desirable changes in the blood levels of glucose and lipids [45,46]. Evidence from RCTs also suggests that supplements of anthocyanins may lower the BMI [45].

Many mechanistic studies have been carried out to investigate how anthocyanins affect the detailed functioning of body systems [47,48,49]. However, there is no reason to believe that the findings from these studies have significantly affected the overall conclusions.

## 7. Mechanistic Research: Lessons Learned

The evidence presented above supports the argument that mechanistic research has been of remarkably little value in revealing which aspects of the diet increase the risk of disease and which are protective. The explanation for this is perfectly simple: the body is immensely complex, and our understanding is so limited that we cannot use findings from mechanistic research to accurately predict how a change in the diet will affect disease risk. Cohort studies and RCTs, by contrast, look directly at how a change in input affects output, while ignoring the “black box” that lies in between.

Nevertheless, mechanistic research still appears to be as popular as ever among researchers in the field of diet and disease. In recent years two new areas have become the focus of mechanistic research with, presumably, the goal of explaining the details of how diet affects the risk of disease. These are chronic inflammation [50,51,52] and the microbiome [53,54,55]. Past experience points to four likely outcomes from this research strategy:

Many interesting findings will be made. For millennia humans have been full of curiosity as to how the natural world works. Learning more about the detailed workings of the human body will generate new knowledge which many people will no doubt find fascinating.

This research may lead to the discovery of new biomarkers. These are potentially valuable but only when there is solid evidence that the level of the biomarker correlates with the risk of a disease, either positively or negatively. Research on chronic inflammation has already led to the discovery of several biomarkers, that appear to be related to the risk of NCD when tissue levels are elevated. Examples include C-reactive protein, interleukin-6 (IL-6), and tumor necrosis factor (TNF) [50].

New therapies, such as drugs, may be discovered. Indeed, we are already seeing early evidence of this with the discovery of probiotics which improve health by bringing about beneficial changes to the microbiome in the colon [56,57,58].

As regards finding effective new dietary treatments for various disorders, the chances of success are low.

## 8. Conclusions

The evidence discussed here demonstrates that several of the most important research methods employed for investigating the relationship between diet, health, and disease are prone to produce misleading findings. The key conclusions from this paper are as follows.

Cohort studies and RCTs each have strengths and limitations. It is an oversimplification of a complex issue to place RCTs above cohort studies in the research hierarchy. The advantages of cohort studies are that in the vast majority of cases they have a much longer period of follow-up. Furthermore, subjects are normally free of the disease of interest when recruited. As a result, the role of diet in relation to risk can be investigated at all stages of the disease etiology. RCTs, by contrast, typically recruit subjects who already have the disease of interest or are at high risk for it. Moreover, the period of follow-up may not be long enough. For these reasons, it is essential to carefully evaluate each RCT based on its design features.

While the use of biomarkers and of other surrogate endpoints have clear advantages in research studies, by including cohort studies and RCTs, they may also be a source of misleading findings. The use of checklists may be a valuable addition to research publications. This paper described the use of CONSORT when reporting the findings of RCTs that use surrogate endpoints. Similarly, STROBE is a checklist for use in observational studies.

Confounding is another important problem that can lead to significant error in observational studies. While this source of potential error is widely recognized, it is not always given proper attention. This issue is discussed in the text.

Reverse causation is another source of possible error in observational studies and may occur if a change in the health-related outcome leads to a change in diet or lifestyle. Cross-sectional studies are especially prone to this problem as they do not allow researchers to determine the temporal sequence of lifestyle and other inputs together with health-related outcomes. The possibility of reverse causation is an additional reason why researchers must always be cautious before concluding that the findings of an observational study indicate a cause-and-effect relationship.

Researchers working in this area therefore need to be cognizant of these potential problems. Furthermore, persons who serve as reviewers for papers also need to consider these issues as do readers of research papers.

Only a small number of potential problems with research methods have been discussed in this paper, and it must be stressed that many other possible problems are likely to be present. Hopefully, others will build on the arguments presented here.

This paper also made the case that mechanistic research has only a very limited value in the areas under discussion here. It is likely that most researchers who carry out mechanistic research would vigorously argue against this assertion. However, those who reject my argument should point to solid evidence where they are right, and I am wrong. Time will tell.

## Data Availability

No new data were created or analyzed in this study.
